# Effect of proton pump inhibitors on the clinical outcomes of PD-1/PD-L1 inhibitor in solid cancer patients

**DOI:** 10.1097/MD.0000000000030532

**Published:** 2022-09-09

**Authors:** Bing Wu, Congcong Sun, Xiaoqin Sun, Xue Li

**Affiliations:** a Department of Oncology, Weihai Cental Hospital, Weihai, China; b Department of Oncology, Weihai Wendeng District People’s Hospital, Weihai, China; c Department of Oncology, Weihai Wendeng District Zetou Township Health Center, Weihai, China; d Department of Clinical Teaching, Weihai Health School, Weihai, China.

**Keywords:** clinical outcome, meta-analysis, programmed death ligand-1 (PD-L1) inhibitor, programmed death protein-1 (PD-1), proton–pump inhibitor (PPI)

## Abstract

Some concomitant drugs may affect the efficacy of programmed death protein-1/ ligand-1 (PD-1/L1) inhibitors. Among the various concomitant drugs, proton–pump inhibitors (PPI) have attracted some attention but have not reached a conclusion. We conducted a meta-analysis to evaluate the impact of PPIs on the survival of cancer patients treated with PD-1/L1 inhibitors.

**Material/methods:**

Related databases and conferences reports were searched. Studies that reported the relationship between PPI use and clinical outcomes of PD-1/L1 inhibitors were included. Meta-analysis was conducted to obtain pooled hazard ratios (HR)s with 95% confidence interval (CI).

**Results:**

Eight studies involving 4869 cancer patients were included. Meta-analysis showed that PPI use was associated with worse overall survival (OS) (HR = 1.43, 95% CI 1.32–1.56), worse progression free survival (PFS) (HR = 1.30, 95% CI 1.20–1.40), and decreased objective response (odds ratio = 0.71, 95% CI 0.58–0.87) in cancer patients receiving PD-1/L1 inhibitors. Neither cancer type nor therapy type affected the effect of concomitant PPIs on the OS and PFS. In the subgroup of studies with a population size <500, PPIs did not reduce the OS, but the PFS. Only 1 single-center study was conducted, showing that PPI use did not affect the OS and PFS. There was no evidence of publication bias among included studies.

**Conclusion:**

Concomitant PPI use was correlated with worse clinical outcomes in cancer patients treated by PD-1/L1 inhibitors. Further prospective clinical and experimental studies are needed to confirm the effect and mechanism of PPI in worsening the clinical outcome of PD-1/L1 inhibitors.

## 1. Introduction

Immunotherapy has made astounding advances in the treatment of cancer.^[1]^ Programmed death protein-1/ligand-1 (PD-1/PD-L1) inhibitors, representative immune checkpoint inhibitors used in immunotherapy,^[1,2]^ have been approved to treat a variety of tumors, such as non-small cell lung cancer (NSCLC), urothelial cancer, melanoma, head and neck squamous cell cancer, lymphoma.^[3]^

As the spectrum of PD-1/PD-L1 inhibitors expands, drug-drug interactions have become a field of particular interest. Some concomitant drugs may enhance or discount the efficacy of immunotherapy,^[4]^ such as antibiotics^[5]^ and corticosteroids.^[6,7]^ Recently, a body of studies have been conducted to analyze the general impact of proton–pump inhibitors (PPIs) on the efficacy of PD-1/PD-L1 inhibitors.

PPIs can irreversibly inhibit the hydrogen-potassium-ATPase. Long-term use of PPIs can change the structure of microbiome.^[8,9]^ Previous studies have proved the that gut microbiota are closely implicated in native immunity. The interaction between PPIs and bPD-1/PD-L1 inhibitors has been explored in some retrospective studies.^[6,7,10–16]^ Unfortunately, no consensus has arrived. Here, we systematically reviewed the literature and made a meta-analysis to explore the impact of PPI use on the outcomes of PD-1/PD-L1 inhibitors in advanced cancer patients.

## 2. Materials and Methods

### 2.1. Search strategy

This study was registered in PROSPERO with registration number CRD42021273673. Relevant literature published before June 1, 2021 were searched by 2 researchers independently from databases and conference proceedings such as EMBASE, PubMed, Cochrane, European Society for Medical Oncology, American or Chinese Society of Clinical Oncology, and American Association for Cancer Research.

We used keywords such as “PD-1 inhibitor,” “PD-L1 inhibitor,” “avelumab,” “atezolizumab,” “camrelizumab,” “nivolumab,” “pembrolizumab,” “sintilimab,” “durvalumab,” “toripalimab,” “sintilimab,” “proton pump inhibitor,” “omeprazole,” “pantoprazole,” “lansoprazole,” “rabeprazole,” “esomeprazole,” “dexlansoprazole,” “esomeprazole.” Literature in any language was reviewed.

### 2.2. Selection and inclusion of studies

We included randomized controlled trials and retrospective trials that met inclusion criteria. Inclusion criteria included: Patients were definitely diagnosed with solid cancer and received PD-1/PD-L1 inhibitor alone or in combination with other anti-cancer drugs; PPIs were prescribed before, during, or after PD-1/PD-L1 inhibitor treatment in the experimental group, but did not prescribe in the control group; Articles reported the clinical outcomes, such as overall survival (OS), progression-free survival (PFS), or objective response rate (ORR). The eligible studies were screened and included by 2 independent investigators.

### 2.3. Data extraction

The following information was extracted from the eligible articles: authors, year of publication, region/country, number of centers, inclusion period, study size, cancer type, treatment line, name of PD-1/PD-L1 inhibitor, treatment type, PPI type, duration of PPI treatment, hazard ratio (HR) (odds ratio) and 95% confidence interval (CI) of OS, PFS, and ORR. Cutoffs in every study was listed in Table 1.

**Table 1:** Baseline characteristics of studies included in this meta-analysis.

Study	Region/country	Center	Inclusion period	Cancer	Line		Combination
Chalabi 2020	31 countries in Europe and North America	Multi-center	Aug 2013-Nov 2014	NSCLC	Second- and higher line		Monotherapy
Cortellini 2021	Italy	Multi-center	Jan 2017-May 2020	NSCLC	First line		Monotherapy
Cortellini 2021-2	Italy	Multi-center	Jun 2014-Mar 2020	pan-cancer	First- or higher line		Monotherapy
Hopkins 2020	Australia	Multi-center	May 2014-Feb 2016	urothelial cancer	First- or second line		Monotherapy
Hopkins 2021	26 countries	Multi-center	Mar 2015-Dec 2016	NSCLC	First line		Combination
Ruiz-Banobre 2021	Spain	Multi-center	Jun 2016-Feb 2020	urothelial carcinoma	First- or higher line		Monotherapy
Svaton 2020	Czech Republic	Multi-center	2015 and 2019	NSCLC	First- or higher line		Monotherapy
Zhao 2019	China	Single-center	Jan 2016-Jan 2018	NSCLC	Any line		Monotherapy or combination
Study	Treatment	Size	PPI type	Period of PPI	Outcome	Cutoff of treatment	
Chalabi 2020	Atezolizumab	757	Omeprazole, pantoprazole,lansoprazole, esomeprazole, rabeprazole, and dexlansoprazole	30 days prior and 30 days after treatment initiation	PFS,OS	July 7, 2016 (OAK trial); May 8, 2015 (POPLAR Trial)	
Cortellini 2021	Pembrolizumab	950	NA	NA	PFS,OS,ORR	September 2020;	
Cortellini 2021-2	Any PD-1/PD-L1 inhibitor	1012	NA	NA	PFS,OS,ORR	May 2020;	
Hopkins 2020	Atezolizumab	896	Omeprazole, pantoprazole, esomeprazole, lansoprazole, rabeprazole, and dexlansoprazole	30 days prior and 30 days after treatment initiation	PFS,OS	July 4 2016 (Imvigor 210); March 13,2017 (Imvigor 211)	
Hopkins 2021	Atezolizumab	802	Omeprazole, pantoprazole, esomeprazole, lansoprazole, rabeprazole, and dexlansoprazole.	30 days prior and 30 days after treatment initiation	PFS,OS	September 15, 2017;	
Ruiz-Banobre 2021	Atezolizumab, pembrolizumab, nivolumab, durvalumab	119	NA	30 days prior and 30 days after treatment initiation	PFS,OS,ORR	February 2020;	
Svaton 2020	Nivolumab	224	Omeprazole, pantoprazole, and lansoprazole	1 month before and 1 month after treatment initiation	PFS,OS	December 2020;	
Zhao 2019	Pembrolizumab, nivolumab, or atezolizumab	109	NA	1 month before and 1 month after treatment initiation	PFS,OS	May 2018;	

NOS = Newcastle-Ottawa Quality Assessment Scale, NSCLC = non-small cell lung cancer, ORR = objective response rate, OS = overall survival, PD-1 = programmed death protein-1, PD-L1 = programmed death ligand-1, PFS = progression free survival, PPI = proton–pump inhibitor.

### 2.4. Quality assessment

The quality of included retrospective studies was evaluated by the Newcastle-Ottawa quality assessment scale.^[17]^ Newcastle-Ottawa quality assessment scale scores were graded by 2 reviewers separately and discussed by all authors of the present study. A score 7 or 8, indicated a high quality.^[18]^

### 2.5. Statistics

Overall HRs with 95% CI for PFS or OS were used to compare the impact of concomitant PPIs on the efficacies of PD-1/PD-L1 inhibitors. The results favor the PPI-using group when the pooled HR <1. Conversely, the results favor the no PPI-using group when the pooled HR >1. *I*^2^ statistics were used to evaluate the heterogeneity, and *I*^2^ > 50% and/or *P* < .10 were judged as heterogeneously. The random effects model was used for pooling heterogeneous studies, and the fixed effects model was adopted for pooling homogeneous studies. Subgroup analysis was made to investigate the influence of cancer type, therapy type, study size, and center number. Publication bias was evaluated by Egger test. Sensitivity analysis was conducted by using the “one-study removed” method. All statistics were made by Stata software (Version 13.0, Stata Corporation, College Station, TX).

## 3. Results

### 3.1. Study selection

**Figure 1.**
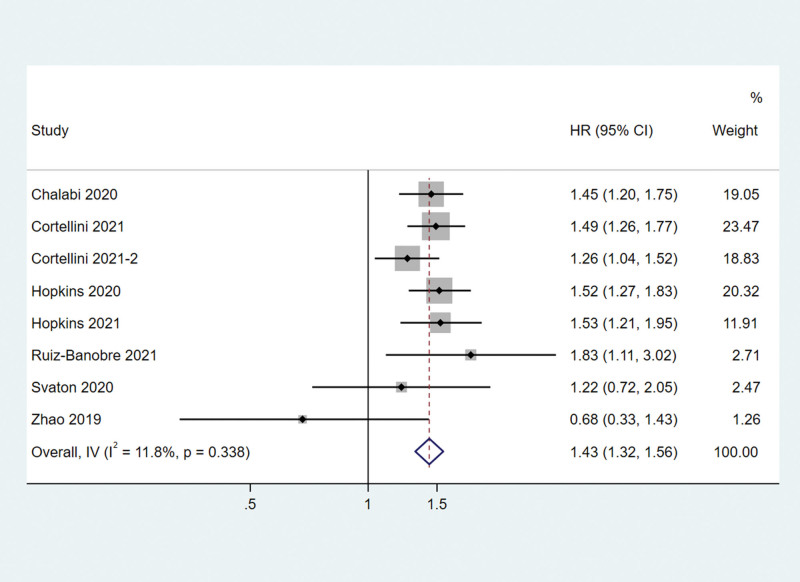
Associations between PPI use and OS in cancer patients treated with PD-1/L1 inhibitors. OS = overall survival, PD-1/L1 = programmed death protein-1/ligand 1, PPI = proton–pump inhibitor.

A flowchart demonstrating how studies were selected and included is shown in Figure 1. A total of 8 articles were included in this meta-analysis (Figure S1, Supplemental Digital Content 1, http://links.lww.com/MD/H297), including 7 were conducted in multi-centers, 5 studies in NSCLC patients, 3 studies using PD-L1 inhibitor Atezolizumab, 6 studies using PD-1/PD-L1 inhibitors monotherapy. All these retrospective studies were graded as 7 or 8 (Table S1, Supplemental Digital Content 2, http://links.lww.com/MD/H298), indicating their high quality. Table 1 shows the characteristics of included studies. The study size ranged from 109 to 1012 patients. Immunotherapy was performed as first-line, second-line, or beyond treatment

Finally, eight studies involving 4869 patients were included. PPIs were given to 1933 patients (39.70%). PPI type was reported in 4 studies, time window of PPI use in 6 studies, PFS and OS in 8 studies, and ORR in 3 studies.

### 3.2. OS and subtype analysis

All the 8 studies reported the impact of PPI use on the OS and PFS of patients receiving PD-1/PD-L1 inhibitors. The meta-analysis showed that PPI use was associated with a shorter OS. PPI use increased the risk of death by 43% (HR = 1.43, 95% CI 1.32–1.56) in solid cancer patients receiving PD-1/PD-L1 inhibitors (Fig. 1). There was no significant heterogeneity among these studies (*I*^2^ = 11.8%, *Q* test *P *= .338).

**Figure 2.**
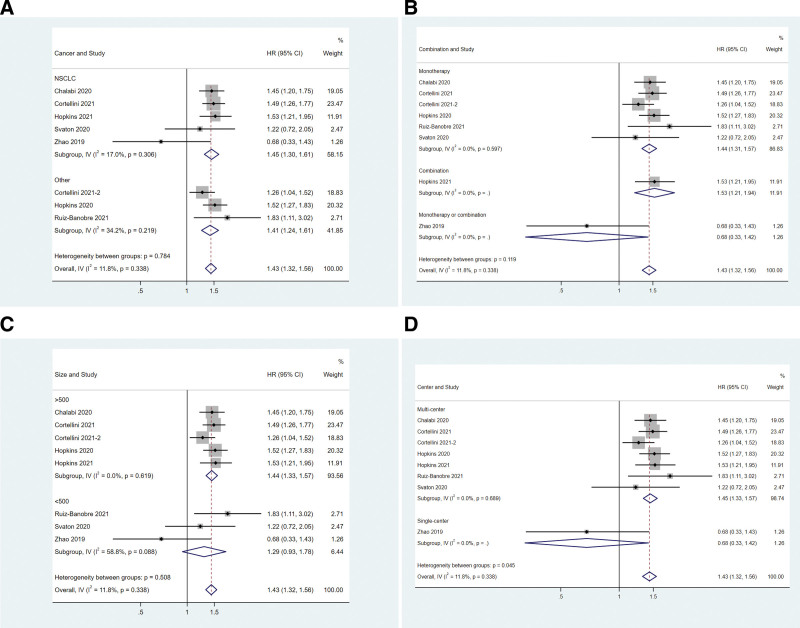
Associations between PPI use and OS in cancer patients stratified by cancer type (A), treatment type (B), study size (C), and center number (D). OS = overall survival, PPI = proton–pump inhibitor

Subgroup analyses were performed based on cancer type, study size, therapy type, and center number. Neither cancer type (Fig. 2A) nor therapy type (Fig. 2B) impacted the effect of concomitant PPIs on OS. The OS of NSCLC patients (HR = 1.45, 95%CI: 1.30–1.61) or others (HR = 1.41, 95%CI: 1.24–1.61) were reduced by concomitant PPI use (Fig. 2A). The OS in either monotherapy (HR = 1.44, 95%CI: 1.31–1.57) or combination therapy (HR = 1.42, 95%CI: 1.13–1.78) subgroup was affected by concomitant PPI use (Fig. 2B). In the subgroup of studies with a size >500, PPI-users had worse OS than non-PPI users (HR = 1.44, 95%CI: 1.33–1.57, Fig. 2C). But in the subgroup of studies with a size <500, PPI-users had similar OS as those of non-PPI users (HR = 1.29, 95%CI: 0.93–1.78, Fig. 2C). In the study of Zhao in 2019, which was conducted in a single center, PPI use did not affect the OS (HR = 0.68, 95%CI: 0.33–1.42, Fig. 2D). The other 7 studies were conducted in multiple centers, and PPI use was associated with a shorter OS (HR = 1.45, 95%CI: 1.33–1.57, Fig. 2D).

### 3.3. PFS and subtype analysis

**Figure 3.**
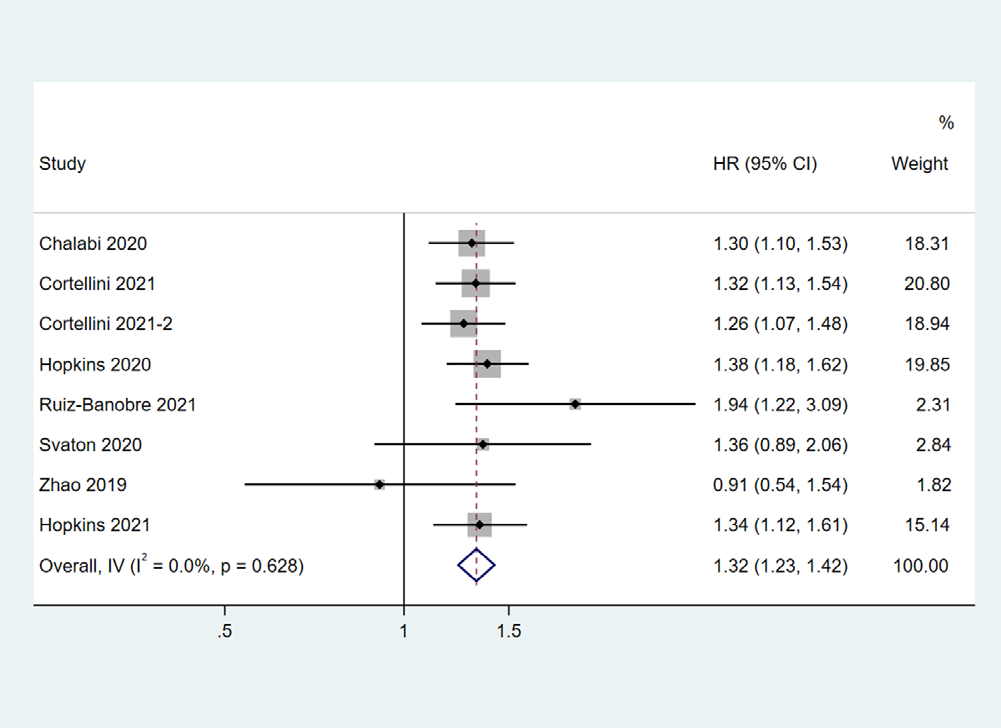
Associations between PPI use and OS in cancer patients treated with PD-1/L1 inhibitors. OS = overall survival, PD-1/L1 = programmed death protein-1/ligand 1, PPI = proton–pump inhibitor.

The meta-analysis showed that PPI use was also associated with shorter PFS in solid cancer patients receiving PD-1/PD-L1 inhibitors. PPI use increased the risk of death by 32% (HR = 1.32, 95% CI 1.23–1.42) (Fig. 3). No statistically significant heterogeneity was observed across these studies (*I*^2^ = 0.0%, *Q* test *P *= .628, Fig. 3).

**Figure 4.**
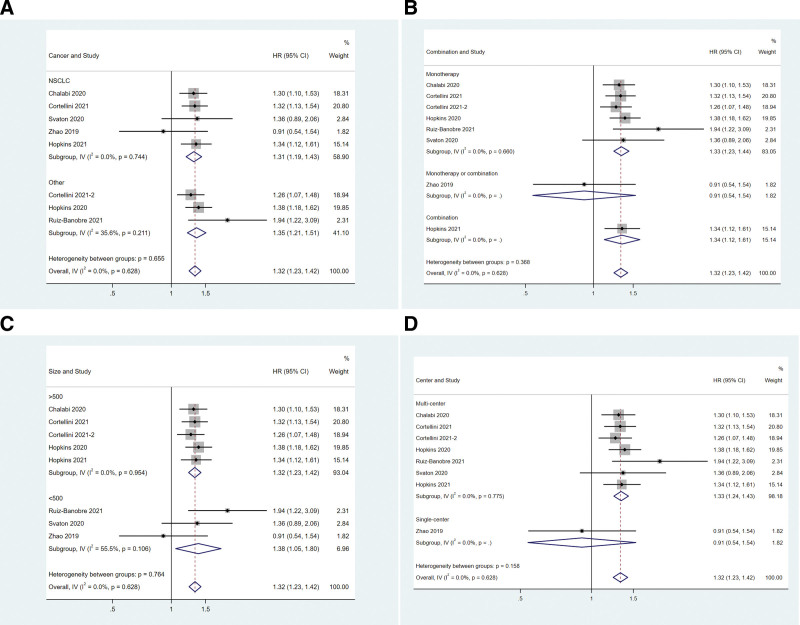
Associations between PPI use and PFS in cancer patients stratified by cancer type (A), treatment type (B), study size (C), and center number (D). PFS = progression free survival, PPI = proton–pump inhibitor.

In the subgroup analysis, concomitant PPIs significantly reduced the PFS in either NSCLC patients (HR = 1.31, 95%CI: 1.19–1.43, Fig. 4A) or other cancer patients (HR = 1.35, 95%CI: 1.21–1.51, Fig. 4A). The OS in either monotherapy (HR = 1.33, 95%CI: 1.23–1.44) or combination therapy (HR = 1.29, 95%CI: 1.08–1.53) subgroup was affected by concomitant PPIs (Fig. 4B). Study size did not impact the effect of concomitant PPIs on PFS (Fig. 4C). In the single-center study “Zhao 2019,” PPIs did not affect the PFS (HR = 0.91, 95%CI: 0.54–1.54, Fig. 4D). The meta-analysis of multi-center studies indicated that PPI use was correlated with shorter PFS in solid cancer patients using PD-1/PD-L1 inhibitors (HR = 1.33, 95%CI: 1.24–1.43, Fig. 4D).

### 3.4. Objective response rate

**Figure 5.**
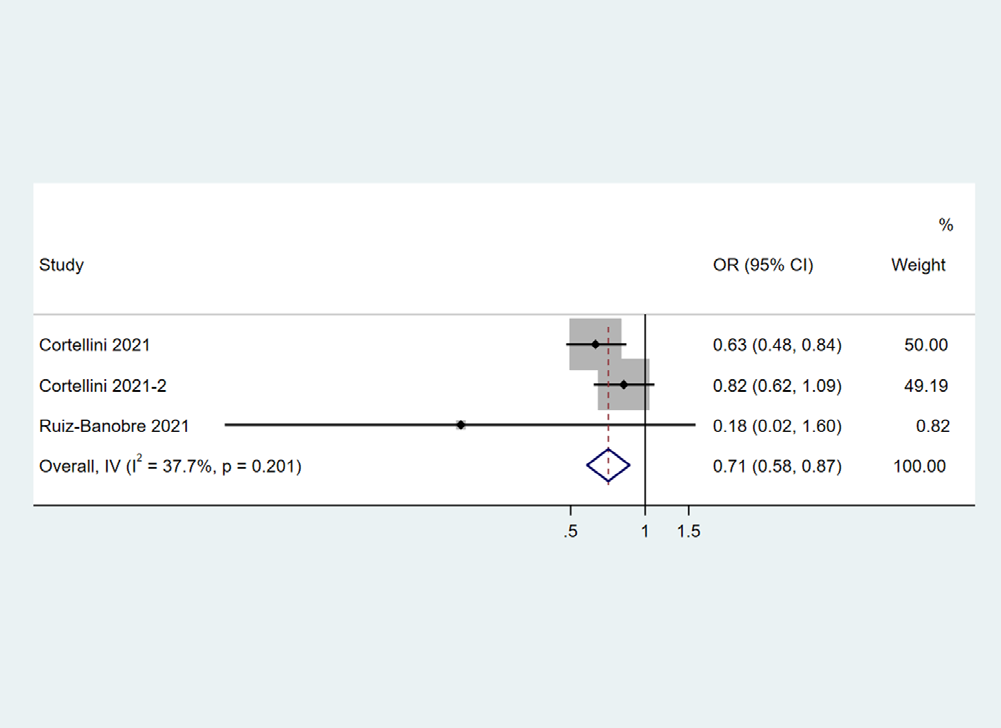
Associations between PPI use and objective response rate in cancer patients treated with PD-1/L1 inhibitors. PPI = proton–pump inhibitor; PD-1/L1 = programmed death protein-1/ligand 1.

Three studies reported ORR. The meta-analysis showed that PPI use was associated with a lower ORR. PPI use decreased the odd rate of response by 29% (odds ratio = 0.71, 95% CI 0.58–0.87) in solid cancer patients receiving PD-1/PD-L1 inhibitors (Fig. 5). There was not significantly heterogeneity among these studies (*I*^2^ = 37.7%, *Q* test *P* = .201).

### 3.5. Publication bias and sensitivity

**Figure 6.**
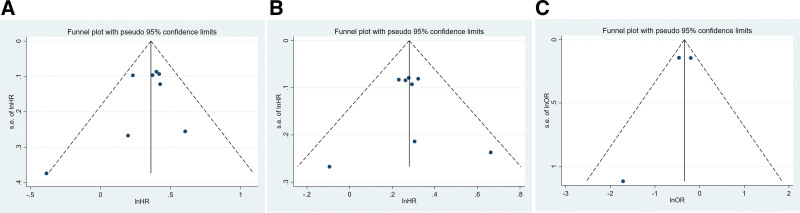
Publication bias analysis of OS (A) and PFS (B). OS = overall survival, PFS = progression free survival.

Egger test showed no publication bias among the 8 studies (*P* = .349 for OS; *P* = .883 for PFS, P = .521 for ORR, Fig. 6A, B, and C). Funnel plots demonstrated symmetry in the HR of either OS or PFS (Figure S2, Supplemental Digital Content 3, http://links.lww.com/MD/H299).

## 4. Discussion

Some concomitant drugs might affect the efficacy of PD-1/PD-L1 inhibitors. In this study, we revealed that PPI users had shorter OS and PFS than non-PPI users in advanced solid cancer patients receiving PD-1/PD-L1 inhibitors. Neither cancer type nor therapy type impacted the efficacies of concomitant PPIs on clinical outcomes.

Three meta-analyses have investigated the impacts of PPIs on immune checkpoint inhibitors (including PD-1/PD-L1 inhibitor and CTLA-4 inhibitor), but got different results.^[8,19,20]^ Subgroup analysis has found that concomitant PPIs may have a positive effect on the prognosis of melanoma patients and a negative effect on the prognosis of NSCLC patients.^[19]^ Further analysis found that melanoma patients mainly used CTLA-4 inhibitors, but NSCLC patients only used PD-1/PD-L1 inhibitors.^[19]^ This might portend the different impact of PPI on effect of PD-1/PD-L1 inhibitors and CTLA-4 inhibitor. Hence, we focused on advanced cancer patients treated with PD-1/PD-L1 inhibitors and found that PPI use is associated with worse PFS or OS.

PPIs not only impact the gastrointestinal PH, but also the cancer/ immunity interface.^[4]^ PPI have diverse effect, such as immune suppression via reducing the level of adhesion molecules expressed by inflammatory cells, and increasing the secretion of pro-inflammatory cytokines.^[21]^ These effects may worsen the clinical outcomes of ICI treatment. PPIs can alter the pH of gastrointestinal tract and further disrupt the composition of gastrointestinal microbiota.^[19,22]^ This enhances the functions of nitrate/nitrite reductase which is involved in cancer development.^[21,23,24]^ Besides, the alpha diversity of the gastrointestinal microbiota can be decreased by PPIs, which discounts the efficacy of immunotherapy.

But some basic research has obtained opposite conclusions. Solid tumors are characterized by a highly acidic microenvironment that may repress anti-cancer immunity. PPIs can neutralize the pH of the tumor microenvironment and indirectly affect the function of cytotoxic T lymphocytes, thus posing a positive impact on the clinical outcomes of immunotherapies.^[25]^ Furthermore, *Helicobacter pylori* infection weakens the efficacy of cancer immunotherapies in preclinical animal models and NSCLC patients.^[26]^ PPIs can inhibit the growth and reduce the viability of HP directly or indirectly by urase^[27]^ or changing pH.^[28]^ In this perspective, PPIs might improve the efficacy of immunotherapies in cancer patients with HP infection. Hence, basic studies are also needed to explore the possible mechanisms underlying the interaction between PPIs and PD-1/PD-L1 inhibitors.

There are some limitations in our study. First, only retrospective studies were included, which might bring about reporting and selection bias. Second, the effect of type, treatment timing, or the course of PPI was not analyzed due to incomplete data. Third, the impact of concomitant antitumor agents or other non-antitumor agents, other than PPIs, have not been evaluated due to the limited information.

## 5. Conclusion

In conclusion, our meta-analysis revealed that PPI use was associated with worse PFS and OS in advanced cancer patients treated by PD-1/PD-L1 inhibitors. This study provided evidence for the relation between PPI use and clinical outcomes of PD-1/PD-L1 inhibitors. However, the present study elucidated neither the causality between PPI use and efficacy of PD-1/PD-L1 inhibitors, nor the underlying mechanisms. Hence, larger prospective studies and basic research are needed to explain how PPI use changes the clinical outcomes of PD-1/PD-L1 inhibitors in solid cancer patients.

## Author contributions

**Data curation:** Bing Wu.

**Formal analysis:** Congcong Sun.

**Project administration:** Xue Li.

**Writing – original draft:** Bing Wu.

**Writing – review & editing:** Xiaoqin Sun.

## Supplementary Material



## References

[1] Fan L Li Y Chen JY. Immune checkpoint modulators in cancer immunotherapy: recent advances and combination rationales. Cancer Lett. 2019;456:23–8. 30959079 10.1016/j.canlet.2019.03.050

[2] Makuku R Khalili N Razi S. Current and future perspectives of PD-1/PDL-1 blockade in cancer immunotherapy. J Immunol Res. 2021;2021:6661406. 33681388 10.1155/2021/6661406 PMC7925068

[3] Chen Y Pei Y Luo J. Looking for the optimal PD-1/PD-L1 inhibitor in cancer treatment: a comparison in basic structure, function, and clinical practice. Front Immunol. 2020;11:1088. 32547566 10.3389/fimmu.2020.01088 PMC7274131

[4] Hussain N Naeem M Pinato DJ. Concomitant medications and immune checkpoint inhibitor therapy for cancer: causation or association? Hum Vacc Immunother. 2021;17:55–61. 10.1080/21645515.2020.1769398 PMC7872020 32574106

[5] Derosa L Hellmann MD Spaziano M. Negative association of antibiotics on clinical activity of immune checkpoint inhibitors in patients with advanced renal cell and non-small-cell lung cancer. Ann Oncol. 2018;29:1437–44. 29617710 10.1093/annonc/mdy103 PMC6354674

[6] Svaton M Zemanova M Zemanova P. Impact of concomitant medication administered at the time of initiation of nivolumab therapy on outcome in non-small cell lung cancer. Anticancer Res. 2020;40:2209–17. 32234916 10.21873/anticanres.14182

[7] Cortellini A Tucci M Adamo V. Integrated analysis of concomitant medications and oncological outcomes from PD-1/PD-L1 checkpoint inhibitors in clinical practice. J ImmunoTher Cancer. 2020;8:e001361. 33154150 10.1136/jitc-2020-001361 PMC7646355

[8] Qin BD Jiao XD Zhou XC. Effects of concomitant proton pump inhibitor use on immune checkpoint inhibitor efficacy among patients with advanced cancer. Oncoimmunology. 2021;10:1929727. 34350061 10.1080/2162402X.2021.1929727 PMC8296970

[9] Rogers MAM Aronoff DM. The influence of non-steroidal anti-inflammatory drugs on the gut microbiome. Clin Microbiol Inf. 2016;22:178.e1–9. 10.1016/j.cmi.2015.10.003 PMC4754147 26482265

[10] Chalabi M Cardona A Nagarkar DR. Efficacy of chemotherapy and atezolizumab in patients with non-small-cell lung cancer receiving antibiotics and proton pump inhibitors: pooled post hoc analyses of the OAK and POPLAR trials. Ann Oncol. 2020;31:525–31. 32115349 10.1016/j.annonc.2020.01.006

[11] Cortellini A Di Maio M Nigro O. Differential influence of antibiotic therapy and other medications on oncological outcomes of patients with non-small cell lung cancer treated with first-line pembrolizumab versus cytotoxic chemotherapy. J ImmunoTher Cancer. 2021;9:e002421. 33827906 10.1136/jitc-2021-002421 PMC8031700

[12] Hopkins AM Kichenadasse G Karapetis CS. Concomitant proton pump inhibitor use and survival in urothelial carcinoma treated with atezolizumab. Clin Cancer Res. 2020;26:5487–93. 32933995 10.1158/1078-0432.CCR-20-1876

[13] Mukherjee S Ibrahimi S Khalid B. Do proton pump inhibitors modulate the efficacy of anti-PD-1/PD-L1 therapy? A retrospective study. J Oncol Pharm Prac. 2019;25:762–4. 10.1177/1078155218771152 29690815

[14] Peng K Chen K Teply BA. Impact of proton pump inhibitor use on the effectiveness of immune checkpoint inhibitors in advanced cancer patients. Ann Pharmacother. 2021;56:377–86. 34282636 10.1177/10600280211033938

[15] Ruiz-Bañobre J Molina-Díaz A Fernández-Calvo O. Rethinking prognostic factors in locally advanced or metastatic urothelial carcinoma in the immune checkpoint blockade era: a multicenter retrospective study. ESMO Open. 2021;6:100090. 33740735 10.1016/j.esmoop.2021.100090 PMC7980066

[16] Zhao S Gao G Li W. Antibiotics are associated with attenuated efficacy of anti-PD-1/PD-L1 therapies in Chinese patients with advanced non-small cell lung cancer. Lung Cancer (Amsterdam, Netherlands). 2019;130:10–7. 30885328 10.1016/j.lungcan.2019.01.017

[17] Zhang Y Huang L Wang D. The ROBINS-I and the NOS had similar reliability but differed in applicability: a random sampling observational studies of systematic reviews/meta-analysis. J Evide Based Med. 2021;14:112–22. 10.1111/jebm.12427 34002466

[18] Stang A. Critical evaluation of the Newcastle-Ottawa scale for the assessment of the quality of nonrandomized studies in meta-analyses. Eur J Epidemiol. 2010;25:603–5. 20652370 10.1007/s10654-010-9491-z

[19] Li M Zeng C Yao J. The association between proton pump inhibitors use and clinical outcome of patients receiving immune checkpoint inhibitors therapy. Int Immunopharmacol. 2020;88:106972. 33182025 10.1016/j.intimp.2020.106972

[20] Li C Xia Z Li A. The effect of proton pump inhibitor uses on outcomes for cancer patients treated with immune checkpoint inhibitors: a meta-analysis. Ann Trans Med. 2020;8:16551655 1655. 10.21037/atm-20-7498 PMC7812177 33490167

[21] Jackson MA Goodrich JK Maxan ME. Proton pump inhibitors alter the composition of the gut microbiota. Gut. 2016;65:749–56. 26719299 10.1136/gutjnl-2015-310861 PMC4853574

[22] Gori S Inno A Belluomini L. Gut microbiota and cancer: how gut microbiota modulates activity, efficacy and toxicity of antitumoral therapy. Crit Rev Oncol Hematol. 2019;143:139–47. 31634731 10.1016/j.critrevonc.2019.09.003

[23] Imhann F Vich Vila A Bonder MJ. The influence of proton pump inhibitors and other commonly used medication on the gut microbiota. Gut Microbes. 2017;8:351–8. 28118083 10.1080/19490976.2017.1284732 PMC5570416

[24] Pierrard J Seront E. Impact of the gut microbiome on immune checkpoint inhibitor efficacy-a systematic review. Curr Oncol (Toronto, Ont). 2019;26:395–403. 10.3747/co.26.5177 PMC6927774 31896938

[25] Nguyen QP Nomura M Matsumoto S. MO3-10-1—the effect of proton pump inhibitors on the efficacy of nivolumab monotherapy in different types of cancer. Ann Oncol. 2019;30:vi115.

[26] Oster P Vaillant L Riva E. *Helicobacter pylori* infection has a detrimental impact on the efficacy of cancer immunotherapies. Gut. 2021,71:457–66. 34253574 10.1136/gutjnl-2020-323392 PMC8862014

[27] Tsuchiya M Imamura L Park JB. *Helicobacter pylori* urease inhibition by rabeprazole, a proton pump inhibitor. Biol Pharm Bull. 1995;18:1053–6. 8535394 10.1248/bpb.18.1053

[28] Saniee P Shahreza S Siavoshi F. Negative effect of proton-pump inhibitors (PPIs) on *Helicobacter pylori* growth, morphology, and urease test and recovery after PPI removal—an in vitro study. Helicobacter. 2016;21:143–52. 26222264 10.1111/hel.12246

