# Supplementary figures and images for: Effect of proton pump inhibitors on the clinical outcomes of PD-1/PD-L1 inhibitor in solid cancer patients
Source: Medicine (Baltimore). 2022 Sep 9;101(36):e30532. doi: 10.1097/MD.0000000000030532 (PMC10980492; doi:10.1097/MD.0000000000030532)

Figure S1 Flowchart of study selection procedure.

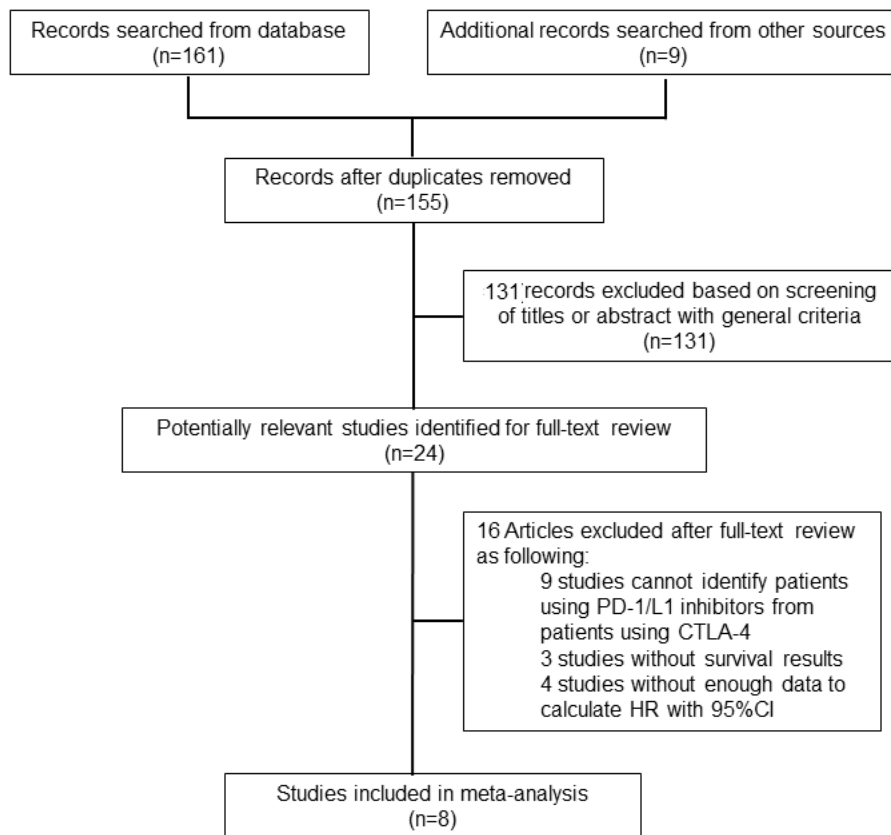

Supplement: Supplementary file 1 [file medi-101-e30532-s001.pdf]

Figure S2 Sensitivity analysis of OS and (A) PFS (B).

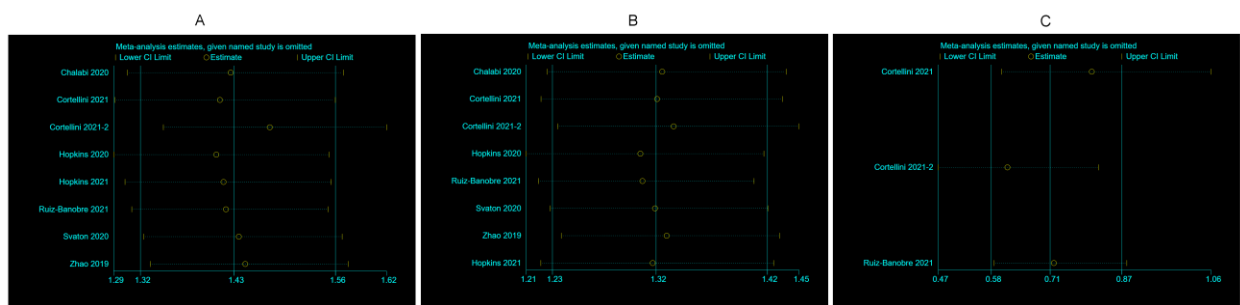

Supplement: Supplementary file 3 [file medi-101-e30532-s003.pdf]
