# Supplementary material for: Effect of proton pump inhibitors on the clinical outcomes of PD-1/PD-L1 inhibitor in solid cancer patients
Source: Medicine (Baltimore). 2022 Sep 9;101(36):e30532. doi: 10.1097/MD.0000000000030532 (PMC10980492; doi:10.1097/MD.0000000000030532)
Supplement: Supplementary file 2 [file medi-101-e30532-s002.pdf]

Table S1 NOS quality assessment of included studies.

Study	Selection[1]				Comparability[2]	Outcome[3]			Score[4]
	A	B	C	D	E	F	G	H	
Chalabi 2020	☆	☆	☆		☆☆	☆	☆	☆	8
Hopkins 2020	☆	☆	☆		☆☆	☆	☆	☆	8
Cortellini 2021	☆	☆	☆		☆☆	☆	☆	☆	8
Cortellini 2021-2	☆	☆	☆		☆☆	☆	☆	☆	8
Svaton 2020	☆	☆	☆		☆☆	☆	☆		7
Peng 2021	☆	☆	☆		☆	☆	☆	☆	7
Ruiz-Bañobre 2021	☆	☆	☆		☆	☆	☆	☆	7
Zhao 2019	☆	☆	☆		☆☆	☆	☆	☆	8

NOS=Newcastle-Ottawa Quality Assessment Scale.
